# Japanese Morals

**Published:** 1890-12

**Authors:** 


### JAPANESE MORALS.

The Japanese ideas of modesty and chastity are very different from ours. If a Japanese woman neglects to hide her charms, it is not from indelicacy as she understands it, but from custom, and the absence of any suspicion that such exposure is indelicate. In this respect they differ from us as much as we do from the Turks, who consider it indelicate and immodest for their women to appear in the streets unveiled. Unchastity in a married woman is by their laws a sufficient ground of divorce, as is also drinking too much, and yet divorces from any or all these causes are not common. If a wife is childless, the husband may divorce her, but she counts it no offense if her husband takes a concubine to raise a family, and she will often, as in patriarchal times, counsel her husband to do so, while she maintains her position as, next to him, the head of the household. Unmarried women and young girls do not account it a vice, nor does the law regard it a crime, to consort temporarily with those of the opposite sex, and they do so without any idea of degradation, but common prostitution is considered degrading. The social evil is under government restriction and surveillance. The Yoshiwara system, as it is there called, places all public houses under government supervision and rigid medical surveillance. It should be added that their patrons are those who have come from Christian countries.

